# Residual Venous Obstruction as an Indicator of Clinical Outcomes following Deep Vein Thrombosis: A Management Study

**DOI:** 10.1055/a-2059-4737

**Published:** 2023-04-12

**Authors:** Aaron F. J. Iding, Bram M. M. Kremers, Alejandro Pallares Robles, Hugo ten Cate, Arina J. ten Cate-Hoek

**Affiliations:** 1 Thrombosis Expertise Center, Heart + Vascular Center, Maastricht University Medical Center, Maastricht, the Netherlands; 2 Department of Biochemistry, Cardiovascular Research Institute Maastricht, Maastricht University, Maastricht, the Netherlands; 3 Center of Thrombosis and Hemostasis, University Medical Center of the Johannes Gutenberg University Mainz, Mainz, Germany; 4 Department of Internal Medicine, Maastricht University Medical Center, Maastricht, the Netherlands

**Keywords:** cancer, deep vein thrombosis, post-thrombotic syndrome, residual venous obstruction, venous thromboembolism

## Abstract

**Background** Residual venous obstruction (RVO) is considered a risk factor of recurrence and possibly other clinical outcomes following deep vein thrombosis (DVT). Current guidelines do not support an RVO-tailored duration of anticoagulant therapy; contemporary data of such management strategies are scarce. We aimed to evaluate an RVO-based management strategy and to assess associations of RVO with recurrence, post-thrombotic syndrome (PTS), arterial events and cancer. To gain further insight, D-dimer levels were measured 1 month after stopping anticoagulant therapy.

**Methods** Consecutive patients with symptomatic, proximal DVT were treated in a 2-year clinical care pathway (CCP) at Maastricht University Medical Center and were followed up to 5 years. RVO was assessed at the end of regular duration of anticoagulant therapy, which was extended once if RVO was detected. The study was approved by the medical ethics committee.

**Result** From a total of 825 patients, 804 patients (97.5%) completed the CCP and 755 (93.9%) were available for extended follow-up. Most patients (76.5%) stopped anticoagulant therapy. Incidence rates of recurrence, PTS, arterial events, and cancer were 4.4, 11.9, 1.7, and 1.8 per 100 patient-years, respectively. RVO was independently associated with PTS (hazard ratio [HR]: 1.66 [1.19–2.32]) and arterial events (HR: 2.07 [1.18–3.65]), but not with recurrence or cancer. High D-dimer was associated with recurrence (HR: 3.51 [2.24–5.48]).

**Conclusion** Our RVO-based management strategy might have attenuated the association of RVO with recurrence. In addition, RVO identified patients at increased risk of PTS and arterial events, which might be used to identify patients in need of alternative treatment strategies.

## Graphical abstract

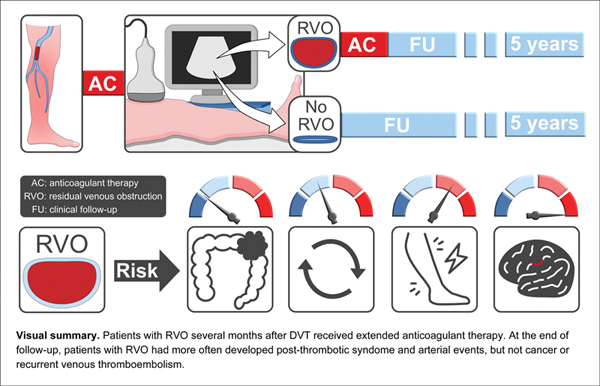

## Introduction

Deep vein thrombosis (DVT) is a common condition that is associated with substantial morbidity and mortality. 1 Its clinical management is mainly focused on recurrence risk reduction and based primarily on anticoagulant therapy. While anticoagulants are indisputably effective in preventing recurrence, the need for extension of initial therapy beyond 3 months remains a subject of debate due to the associated bleeding complications, healthcare costs, and patient burden including repetitive nuisance bleeds. 2 3 Current guidelines recommend not to extend therapy in patients with provoked DVT, but to extend therapy indefinitely in those with unprovoked DVT. 4 5 6 7 This crude dichotomy of therapy duration does not fully appreciate risk differences among patients and might result in inadequately short or unnecessary long therapy. 8 Accordingly, clinicians are known to often deviate from these recommendations in patient management. 9 10 11

Evidently, it is warranted to explore alternative strategies that further stratify patients' risk for recurrence and allow them to stop anticoagulant therapy after tailored duration at acceptable recurrence rates. Such stratification could be based on residual venous obstruction (RVO), which is persistence of thrombotic material detected several months after DVT. RVO has been associated with a significant recurrence risk and attenuation of risk by extended anticoagulant therapy has been assumed. 12 13 However, contemporary data on the applicability and efficacy of a tailored therapy duration based on RVO in clinical practice is scarce.

With management focused on recurrent venous thromboembolism (VTE), other important clinical outcomes in DVT patients might be overlooked. Post-thrombotic syndrome (PTS) remains the most common chronic complication of DVT, and evidence-based prevention is disputably limited to elastic compressive therapy (ECT). 14 15 Arterial events and cancer are also reported to be increased following DVT, although their causal relation remains disputed. 16 17 Interestingly, there have been reports in literature on the association of RVO with these clinical outcomes, particularly with PTS. 18 19 While the pathogenic mechanisms underlying these associations are unclear, a recent study suggests that the presence of RVO could inform the decision to continue long-term ECT to prevent PTS. 20 By extension, perhaps an RVO-based management strategy should be applied to these outcomes as well.

Based on the compelling data published on associations of RVO with recurrent VTE, an RVO-based management strategy was introduced at the outpatient clinic of Maastricht University Medical Center. 21 22 In the current analysis, we aimed to evaluate this management strategy and extend on existing knowledge through an in-depth evaluation of RVO's association with recurrent VTE and other clinical outcomes following DVT. Further insight was obtained by measuring D-dimer levels 1 month after stopping anticoagulant therapy.

## Methods

### Study Design

Consecutive patients diagnosed with symptomatic, proximal DVT of the lower extremity were enrolled in a clinical care pathway (CCP) between July 2003 and December 2018. A detailed description of the CCP can be found elsewhere. 23 In brief, patients had a prospective structured follow-up with regular visits at 0.5, 3, 6, 12, and 24 months after diagnosis, and were treated according to a strict RVO-based management strategy to tailor anticoagulant therapy duration. Upon completing the CCP, patients were instructed to contact the clinical center in case of symptoms suggestive of recurrent VTE. For the current analysis, prospective Ffollow-up data for all clinical outcomes (except PTS) beyond 2 years was extended up to 5 years based on retrospectively gathered data from hospital records. This study was performed in accordance with the Declaration of Helsinki. Study protocol and data collection were approved by the medical ethics committee (METC 15–4-256).

### Management Strategy

Patients were categorized as having “provoked DVT” in the presence of any transient provoking factors within 2 months of the index DVT: surgery or major trauma, long-distance travel (> 10 hours), immobilization (≥ 3 days), hormonal estrogen therapy, pregnancy, or puerperium. All other patients were considered to have “unprovoked DVT.” Regular anticoagulant therapy was 3 or 6 months for provoked or unprovoked DVT, respectively. RVO was assessed 1 week before completion of regular therapy, and if present anticoagulation was extended to twice the regular duration (i.e., 6 or 12 months, respectively). D-dimer was measured 1 month after stopping anticoagulants, and if levels were high (≥500 ng/mL) the option to resume anticoagulant use indefinitely was discussed with the patient. Anticoagulant therapy was never stopped in patients with “high recurrence risk” based on persistent provoking factors (e.g., cancer), unprovoked DVT with previous VTE, or other indications for anticoagulation (e.g., atrial fibrillation).

### RVO Assessment

Compressive ultrasound examination of popliteal, femoral, and common femoral veins was performed by radiologists blinded for patient outcomes. RVO was defined according to the common definition of more than2 mm transversal vein diameter. 21 24 This protocol was found to have acceptable accuracy and interobserver reproducibility. 25 26

### Clinical Outcomes

Recurrent VTE was the primary outcome and included proximal or distal DVT, upper extremity DVT, pulmonary embolism (PE), or unusual site VTE. PTS was diagnosed at least 6 months after DVT when the Villalta score was more than or equal to 5 at two consecutive visits. Arterial events were ischemic stroke, myocardial infarction, coronary revascularization, or peripheral arterial thrombosis. Cancer diagnosis excluded basal or squamous cell skin cancer. Mortality was recorded using last available date and the most likely cause was registered if available.

### Statistical Analysis

Patient characteristics were compared by Mann–Whitney U test, χ ^2^ test, or Fisher's exact test as appropriate. Variables with more than 5% missing values were imputed by random forest imputation using R package “missForest.” The primary outcome of interest was recurrent VTE. A post hoc sample size calculation indicated that 611 patients that stopped anticoagulant treatment would be needed to assess the difference in proportion of recurrent VTE between patients with (group A) and without RVO (group B) at 80% power. 27 Proportions (P _A_ = 15.1%, P _B_ = 9.6%) and sampling ratio (n _A_ /n _B_ = 0.86) were based on data from a systematic review. 12 Incidences were visualized by Kaplan–Meier plots using R version 3.5.3. Patients were censored at death, loss to follow-up or December 2020, whichever came first. Hazard ratios (HR) with 95% confidence interval (CI) were calculated by Cox proportional hazards regression, adjusted for anticoagulant therapy duration and relevant patient characteristics identified by backward stepwise method; D-dimer was also adjusted for age and RVO. Sensitivity analyses were performed by excluding patients with active cancer at baseline and patients with previous ipsilateral DVT. P-values were two-sided and significant if less than 0.05. Analyses were performed in SPSS version 21.0 (SPSS Inc., Chicago, IL). See the Supplementary Material (available in the online version) for further details.

## Results

### Patients and Follow-Up

The cohort consisted of 825 patients with proximal DVT. Patients with unprovoked DVT ( *n* = 472; 57.2%) were significantly older, more often male, had previous VTE, hypertension, and hypercholesterolemia ( Table 1 ). Clinical characteristics had few to no missing values, except for body mass index that had 6.3% missing and was imputed. A total of 804 patients (97.5%; 21 [2.5%] lost to follow-up) completed the CCP and 755 (93.9%; 49 [6.1%] lost to follow-up) patients were available for extended follow-up. Median follow-up duration was 5.0 (3.8–5.0) years, which equates to 3503 patient-years in total.

**Table 1:** Baseline characteristics of study patients

	Total cohort ( *n* = 825)	Provoked DVT ( *n* = 353)	Unprovoked DVT ( *n* = 472)	*p* -Value
Demographic characteristics				
Age in years, median (IQR)	60 (47–72)	52 (42–66)	65 (54–74)	<0.001 a
Male, *n* (%)	424 (51.4)	112 (31.7)	312 (66.1)	<0.001 a
BMI in kg/m ^2^ , median (IQR)	27 (24–30)	28 (24–30)	27 (25–30)	0.984
Clinical characteristics				
Iliofemoral DVT, *n* (%)	202 (24.5)	90 (25.5)	112 (23.7)	0.559
Bilateral DVT, *n* (%)	8 (1.0)	3 (0.8)	5 (1.1)	0.761
Previous VTE, *n* (%)	145 (17.6)	41 (11.6)	104 (22.0)	<0.001 a
Family history of VTE, *n* (%)	236 (29.5)	104 (30.2)	132 (28.9)	0.693
Smoking, *n* (%)	198 (24.4)	83 (24.0)	115 (24.7)	0.821
Venous insufficiency, *n* (%)	87 (10.5)	29 (8.2)	58 (12.3)	0.059
Comorbidities				
Active cancer, *n* (%)	24 (2.9)	11 (3.1)	13 (2.8)	0.760
Hematologic disease, *n* (%)	18 (2.2)	7 (2.0)	11 (2.3)	0.735
Diabetes mellitus, *n* (%)	57 (6.9)	24 (6.8)	33 (7.0)	0.914
Chronic heart failure, *n* (%)	16 (1.9)	4 (1.1)	12 (2.5)	0.146
Cardiovascular risk factors				
Hypertension, *n* (%)	253 (30.7)	85 (24.1)	168 (35.6)	<0.001 a
Hypercholesterolemia, *n* (%)	119 (14.4)	38 (10.8)	81 (17.2)	0.010 a
Antiplatelet drug use, *n* (%)	85 (10.3)	34 (9.6)	51 (10.8)	0.583
Transient provoking factors				
Surgery or major trauma, *n* (%)	–	155 (43.9)	–	–
Long-distance travel, *n* (%)	–	52 (14.7)	–	–
Immobilization, *n* (%)	–	90 (25.5)	–	–
Pregnancy or puerperium, *n* (%)	–	11 (3.1)	–	–
Estrogen therapy, *n* (%)	–	127 (36.0)	–	–
Multiple factors, *n* (%)	–	71 (20.1)	–	–
Anticoagulant therapy				
Vitamin K antagonist, *n* (%)	677 (82.6)	289 (82.3)	388 (82.7)	0.902
Direct oral anticoagulant, *n* (%)	101 (12.3)	46 (13.1)	55 (11.7)	0.550
LMWH, *n* (%)	42 (5.1)	16 (4.6)	26 (5.5)	0.528

Abbreviations: BMI, body mass index; DVT, deep vein thrombosis; IQR, interquartile range; LMWH, low molecular weight heparin; VTE, venous thromboembolism.

a Thrombophilia markers were not routinely ordered and are therefore not reported.

**Fig. 1:**
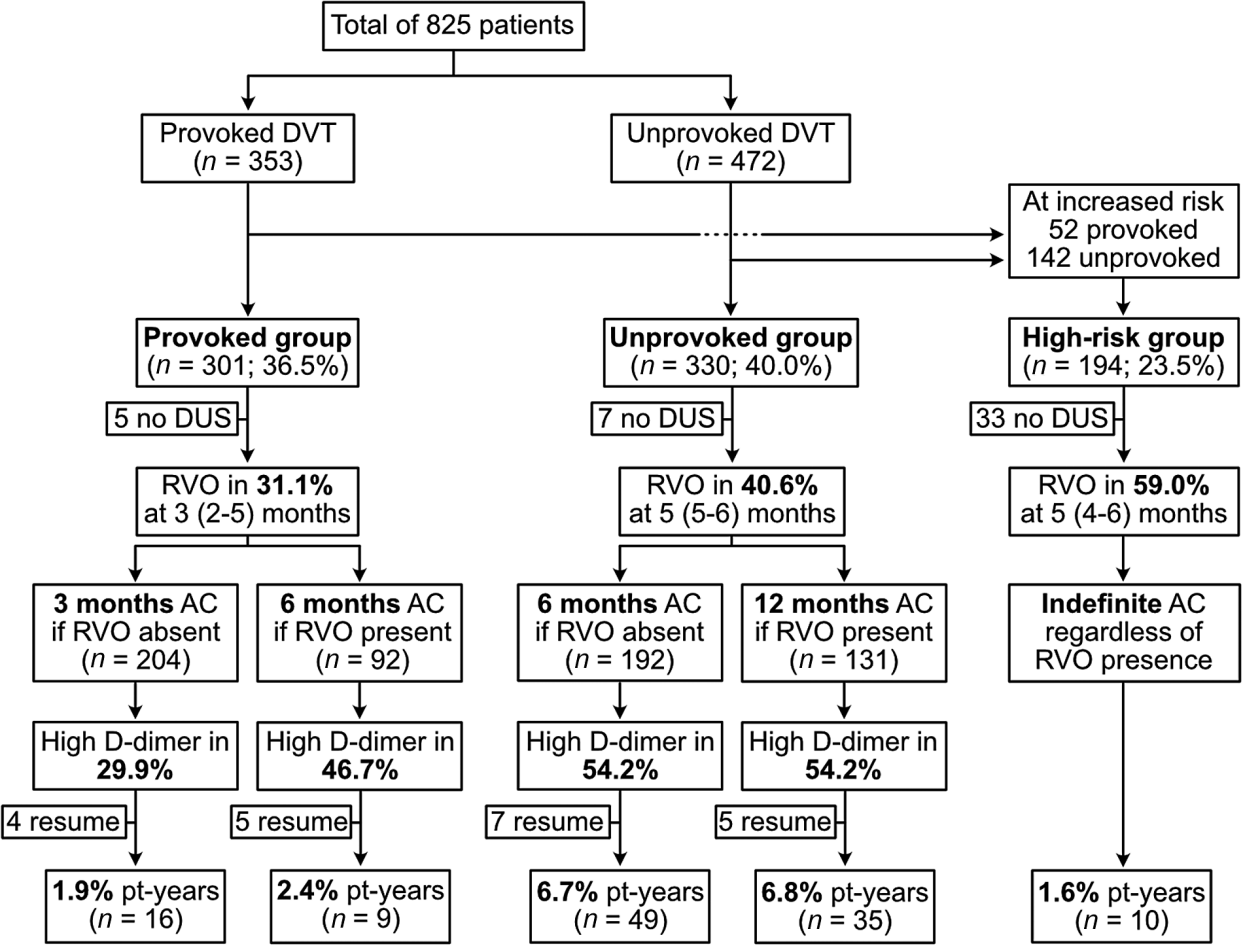
Flow diagram of study design in relation to recurrence rates. Patients were stratified into three groups according to international guidelines. Further stratification was based on the presence of residual venous obstruction (RVO) on duplex ultrasound (DUS) at median time point (interquartile range). Anticoagulant therapy (AC) duration was tailored based on this stratification. One month after stopping anticoagulants, D-dimer levels were measured and considered high at levels >500 ng/mL. Then, patients could resume AC based on their preference. Recurrence rates over 5-year follow-up are shown.

A flow diagram is provided to show the results of the management strategy ( Fig. 1 ). Patients at high recurrence risk ( *n* = 194) were distinguished from other patients with provoked ( *n* = 301) or unprovoked DVT ( *n* = 330). The former group received indefinite anticoagulant therapy, while the latter two groups ( *n* = 631; 76.5%) could stop after RVO-based tailored duration. RVO was not assessed in 45 patients (5.5%) due to various reasons, including 33 patients with indefinite anticoagulation, and those meant to have a tailored duration ( *n* = 12) instead received regular duration. Clinical outcome rates that are reported here concern only those with assessment of RVO ( *n* = 780).

Median durations of anticoagulant therapy were 4.2 months in patients with provoked DVT without RVO, 6.6 months in provoked DVT with RVO, 6.4 months in unprovoked DVT without RVO, and 11.9 months in unprovoked DVT with RVO. One month after tailored anticoagulant therapy, only 21 of 619 patient (3.4%) resumed anticoagulants indefinitely due to high D-dimer, as most patients preferred not to resume balancing the risks during the process of shared decision making. Later during follow-up, 23 patients (4.0%) resumed anticoagulation indefinitely due to newly diagnosed atrial fibrillation ( *n* = 13), antiphospholipid syndrome ( *n* = 2), or other afflictions ( *n* = 8). Besides, 12 patients received anticoagulant therapy for a short period of time due to superficial vein thrombosis.

### Recurrence Rates

Over 5 years of follow-up, 109 patients (17.6%) that stopped anticoagulant therapy had recurrent VTE, corresponding to a rate of 4.4 (3.6–5.3) per 100 patient-years ( Table 2 ). Patients with unprovoked DVT had threefold higher incidence of recurrence than those with provoked DVT. These events included 54 ipsilateral DVTs (49.5%), 20 contralateral DVTs (18.4%), 2 upper extremity DVTs (1.8%), 13 PEs with DVT (11.9%), and 20 PEs without confirmed DVT (18.4%). No unusual site VTEs were observed. In the high recurrence risk group on indefinite anticoagulant therapy, 10 patients (6.2%) had recurrent VTE, attributable to inadequate anticoagulation in 1 patient and newly diagnosed cancer in the others.

**Table 2:** Incidence rates of clinical outcomes

	Total ( *n* = 780)	Provoked DVT ( *n* = 335)		Unprovoked DVT ( *n* = 445)	
		RVO absent ( *n* = 218)	RVO present ( *n* = 117)	RVO absent ( *n* = 201)	RVO present ( *n* = 244)
**Recurrent VTE**					
No. of events (%)	109/619 (17.6)	16/204 (7.8)	9/92 (9.8)	49/192 (25.5)	35/131 (26.7)
Per 100 pt-y (95% CI)	4.4 (3.6–5.3)	1.9 (1.1–3.1)	2.4 (1.1–4.4)	6.7 (5.0–8.8)	6.8 (4.8–9.3)
**PTS**					
No. of events (%)	158 (20.3)	24 (11.0)	34 (29.1) a	43 (17.6)	57 (28.4) ^c^
Per 100 pt-y (95% CI)	11.7 (10.1–13.6)	6.0 (3.9–8.9)	17.5 (12.5–23.6)	10.1 (7.4–13.3)	17.3 (13.4–21.8)
**Arterial event**					
No. of events (%)	53 (6.8)	7 (3.2)	10 (8.5) ^b^	15 (6.1)	21 (10.4)
Per 100 pt-y (95% CI)	1.6 (1.2–2.1)	0.8 (0.3–1.6)	2.1 (1.0–3.9)	1.5 (0.8–2.4)	2.5 (1.6–3.8)
**Cancer**					
No. of events (%)	55 (7.1)	10 (4.6)	6 (5.1)	17 (7.0)	22 (10.9)
Per 100 pt-y (95% CI)	1.7 (1.3–2.2)	1.1 (0.9–2.8)	1.2 (0.6–2.9)	1.7 (1.5–3.5)	2.6 (2.0–4.5)

Abbreviations: CI, confidence interval; DVT, deep vein thrombosis; PTS, post-thrombotic syndrome; pt-y, patient-years; RVO, residual venous obstruction; VTE, venous thromboembolism.

Note: Outcomes are reported for patients with RVO assessment ( *n* = 780/825). Incidences of recurrent VTE are displayed for patients that stopped anticoagulant therapy ( *n* = 619/780), thus excluding “high recurrence risk” patients. PTS was assessed during 2-year follow-up.

a *p* < 0.001 and ^b^ *p* = 0.034 vs provoked DVT with RVO absent; ^c^ *p* = 0.007 vs. unprovoked DVT with RVO absent.

### Rates of Other Outcomes

PTS was diagnosed in 158 patients (20.3%), corresponding to 11.7 (10.1–13.6) per 100 patient-years ( Table 2 ). Arterial events occurred in 53 patients (6.8%), corresponding to 1.6 (1.2–2.1) per 100 patient-years ( Table 2 ). These included 29 ischemic strokes (54.7%), 11 myocardial infarctions (20.8%), 7 coronary revascularizations (13.2%), and 6 peripheral thrombotic events (11.3%). Cancer was diagnosed in 55 patients (7.1%), corresponding to 1.7 (1.3–2.2) per 100 patient-years ( Table 2 ). These included 19 urogenital cancers (34.5%), 8 gastrointestinal cancers (14.5%), 7 lung cancers (12.7%), 7 breast cancers (12.7%), 5 melanomas (9.1%), 4 hematologic cancers (7.3%), 2 brain cancers (3.7%), 2 cancers of unknown primary origin (3.7%), and 1 liposarcoma (1.8%). There was no significant difference in incidences of PTS, arterial events, and cancer diagnosis between patients with unprovoked and provoked DVT ( Table 2 ).

A total of 56 patients died during follow-up, corresponding to a mortality rate of 1.7 per 100 patient-years. No fatal episodes of recurrent VTE or arterial events were recorded. The cause of death was cancer in 22 patients (39.3%), infectious disease in 5 patients (8.9%), various other causes in 8 patients (14.3%), and unknown in 21 patients (37.5%). During follow-up, 9 patients (1.2%) had a major bleeding event while on anticoagulant therapy.

### Associations with RVO

**Fig. 2:**
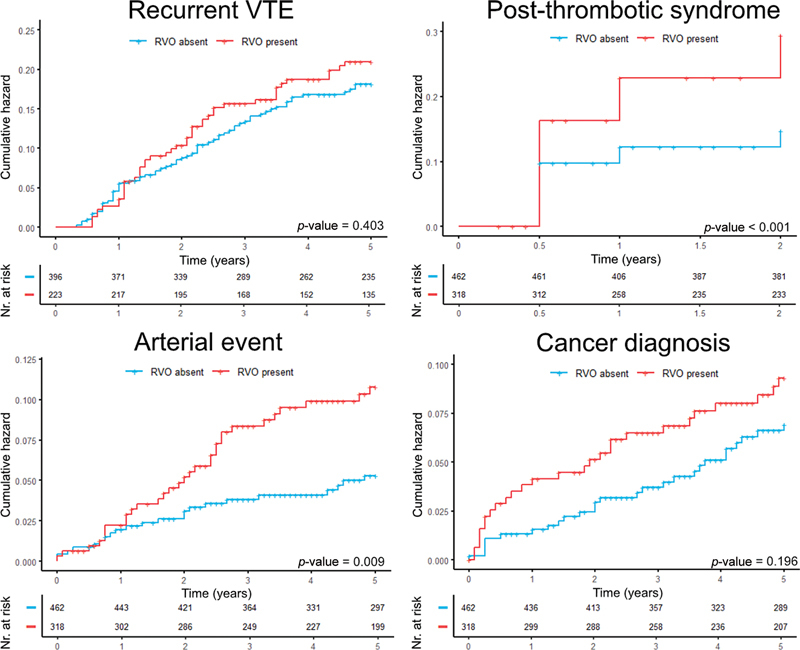
Kaplan–Meier curves of clinical outcomes. Rates of clinical outcomes stratified by residual venous obstruction (RVO). Recurrent venous thromboembolism (VTE) is shown only for patients that stopped anticoagulant therapy, thus excluding the high recurrence risk group. Post-thrombotic syndrome was only assessed during 2-year follow-up. *p* -Values are derived from log-rank tests. Baseline was the moment when deep vein thrombosis was diagnosed.

Overall, RVO was present in 318 of 780 patients (40.8%). Its prevalence increased from provoked to unprovoked patients and was highest in high recurrence risk patients ( Fig. 1 ). Patients with RVO had more than 1.5-fold and 2-fold increased risk of PTS and arterial events, respectively, but no association was found with recurrent VTE or cancer ( Table 3 ). Notably, RVO was strongly associated with PTS in patients with provoked DVT with assessment at 3 months. Differences in incidence rates over time for each outcome were visualized as Kaplan–Meier curves ( Fig. 2 ). Sensitivity analyses for patients with active cancer at baseline (2.1%, *n* = 16/780) or those with previous ipsilateral DVT (8.5%, *n* = 66/780) showed similar results.

**Table 3:** Associations of residual venous obstruction with clinical outcomes

	Total	Provoked DVT	Unprovoked DVT
Recurrent VTE, HR (95% CI)	1.39 (0.96–2.02)	1.50 (0.67–3.37)	1.37 (0.90–2.08)
PTS, HR (95% CI)	1.66 (1.19–2.32) a	2.16 (1.22–3.83) a	1.41 (0.93–2.12)
Arterial event, HR (95% CI)	2.07 (1.18–3.65) a	2.25 (0.82–6.17)	1.98 (1.01–3.92) a
Cancer, HR (95% CI)	1.26 (0.74–2.17)	0.86 (0.31–2.40)	1.48 (0.77–2.82)

Abbreviations: CI, confidence interval; DVT, deep vein thrombosis; HR, hazard ratio; PTS, post-thrombotic syndrome; VTE, venous thromboembolism.

Note: Each association was adjusted for duration of anticoagulant therapy. Additionally, recurrent VTE was adjusted for unprovoked DVT, previous VTE, venous insufficiency and hypertension; PTS was adjusted for body mass index, venous insufficiency, iliofemoral DVT and smoking; arterial events were adjusted for hypertension, antiplatelet drug use and smoking; cancer was adjusted for age and hypertension.

a *p* -Value <0.05.

Several patient characteristics were associated with clinical outcomes. Recurrent VTE was associated with unprovoked DVT (HR: 3.59 [2.29–5.63]) and hypertension (HR: 1.61 [1.11–2.33]). PTS was associated with body mass index (HR: 1.07 [1.04–1.09]), venous insufficiency (HR: 2.41 [1.65–3.53]), iliofemoral DVT (HR: 1.46 [1.04–2.06]), and smoking (HR: 1.57 [1.11–2.21]). Arterial events were associated with hypertension (HR: 2.02 [1.11–3.66]) and antiplatelet drug use (HR: 2.57 [1.31–5.03]). Finally, cancer was associated with age (HR: 1.05 [1.03–1.08]) and hypertension (HR: 2.70 [1.51–4.83]). While risk of recurrent VTE was higher after stopping anticoagulant therapy (HR: 18.94 [7.28–49.32]), no difference was found for other outcomes.

### Associations with Persistent RVO

Upon completion of extended anticoagulant therapy, patients were invited to reassess the presence of RVO without management consequences, to provide reference images in case recurrent VTE was suspected in the future. RVO was reassessed in 152 of 223 patients (68.2%) at median 11 (7–13) months. RVO was still present in 89 of 152 patients (58.6%). However, persistent RVO was neither associated with recurrent VTE (HR: 0.83 [0.39–1.75]), nor with other clinical outcomes (data not shown).

### Associations with High D-Dimer

D-dimer levels were missing and imputed in 12.1% ( *n* = 75) of patients that stopped anticoagulant therapy following tailored duration based on RVO. High D-dimer was found in 279 patients (45.1%) with lowest prevalence in provoked patients without RVO and highest in unprovoked patients irrespective of RVO ( Fig. 1 ). High D-dimer was associated with 3.5-fold increased risk of recurrent VTE, but not with other clinical outcomes ( Table 4 ).

**Table 4:** Associations of high D-dimer with clinical outcomes

	Total	Provoked DVT	Unprovoked DVT
Recurrent VTE, HR (95% CI)	3.51 (2.24–5.48) a	4.56 (1.78–11.68) a	3.16 (1.91–5.24) a
PTS, HR (95% CI)	1.43 (0.93–2.20)	1.46 (0.76–2.79)	1.30 (0.72–2.34)
Arterial event, HR (95% CI)	1.04 (0.50–2.17)	1.01 (0.27–3.80)	0.94 (0.39–2.28)
Cancer, HR (95% CI)	0.67 (0.30–1.49)	0.32 (0.08–1.23) a	1.00 (0.42–2.37)

Abbreviations: CI, confidence interval; DVT, deep vein thrombosis; HR, hazard ratio; PTS, post-thrombotic syndrome; VTE, venous thromboembolism.

Footnote: D-dimer levels were considered high at ≥ 500 ng/mL. Each association was adjusted for duration of anticoagulant therapy, age, and residual venous obstruction. Additionally, recurrent VTE was adjusted for unprovoked DVT, previous VTE, venous insufficiency and hypertension; PTS was adjusted for body mass index, venous insufficiency, iliofemoral DVT and smoking; arterial events were adjusted for hypertension, antiplatelet drug use and smoking; cancer was adjusted for hypertension.

a *p* -Value <0.05.

## Discussion

We evaluated a management strategy with RVO-based tailored duration of anticoagulant therapy. Based on our strategy 3-fourth of DVT patients stopped anticoagulant therapy after tailored duration with an overall recurrence rate of 4.4 per 100 patient-years. Our overall recurrence rate is comparable to rates reported by two earlier studies with tailored anticoagulant therapy duration based on RVO (4.0 and 3.6 per 100 patient-years). 28 29 Both studies included serial D-dimer measurement, which led to many patients being invited to resume anticoagulant therapy. Notably, these studies reported high refusal rates (23 and 8%, respectively), highlighting the underappreciated role of patient preference in current strategies. 4 5 6 7 In our study, even fewer patients (3.4%) resumed anticoagulants following D-dimer measurement as this decision was based on patient preference guided by shared decision making.

Our study, as well as cited studies, contained mostly patients using vitamin K antagonists (VKAs), and the perceived burden of regular laboratory monitoring may have influenced the willingness of patients to resume anticoagulant therapy. The barriers to resume therapy might be lower in patients using direct oral anticoagulants (DOACs). Therefore, the use of D-dimers for risk-stratification could be more relevant in current clinical care. After all, even with DOACs, the burden of indefinite therapy is still considerable.

Observational studies have consistently found RVO to be a predictor of recurrence, although the associations found in later studies were not as strong as the two to five times increased risk found in initial studies. 12 13 21 22 This can be explained by heterogeneity in study design, such as earlier assessment of RVO (i.e., 3–6 months) providing a stronger association. 24 In our management study, we did not find a significant association of RVO with recurrent VTE anymore. In the light of previous findings that observed a reduced recurrence risk by extending anticoagulant therapy in patients with RVO, 30 31 it is plausible that the association in our cohort was attenuated by extending anticoagulant therapy in patients with RVO.

The increased risk of recurrent VTE in patients with RVO is presumed to be caused by a systemic hypercoagulable state, potentially driven by local thromboinflammation. 21 22 32 This hypercoagulable state seems to be transient since recurrence rates decrease over time, advocating the benefit of extended anticoagulant therapy. Nevertheless, based on the persistent association with high D-dimer in our study, it is likely that hypercoagulability continues to have a role after stopping extended anticoagulation as a predictor independent of RVO.

RVO in our cohort proved to be an important indicator of risk for both PTS and arterial events. Several studies found RVO to be associated with a two-times increased risk of PTS, 18 19 while the one study that assessed its relation to arterial events also found a two-times increased risk. 19 Additionally, the latter study found a three-times higher risk of cancer in patients with RVO, which our study did not show. This lack of association is probably due to differences in clinical setting and study population.

RVO's association with PTS is likely explained by excessive inflammation and underlying vein damage instead of hypercoagulability. 33 34 Inflammation is considered to have a major pathogenic role in PTS by contributing to failure of early DVT resolution. 35 36 37 38 39 The importance of early DVT resolution is supported by our observation that PTS was more strongly associated with RVO at 3 compared with 6 months and was not associated with persistent RVO at reassessment. A pathogenic role for coagulation is unlikely since PTS was neither associated with duration of anticoagulant therapy nor with high D-dimer. 40 41 42

The association of RVO with arterial events points to an often proposed mechanistic “crosstalk” between the venous and arterial vasculature. 35 While hypercoagulability would be expected to play a role, 19 we did not observe a protective effect of extended anticoagulant therapy, in agreement with one study. 36 Another large cohort, however, reported reduced a risk of arterial events in those with anticoagulant treatment beyond 3 months, meaning a role of hypercoagulability cannot be ruled out. 37 38 Additionally, this risk of arterial events in VTE might be explained by thrombus persistence and atherogenesis having common pathogenic mechanisms, including the role of proinflammatory macrophages. 39 Indeed, subclinical atherosclerosis was found to be almost three times as prevalent in patients with RVO as compared with those without RVO. 43 While the finding of DVT in any patient should trigger revision of cardiovascular risk factors, the finding of RVO should be considered an additional important risk indicator for arterial events. Moreover, in the context of the currently increasing use of reduced-dose DOACs for secondary prevention of VTE, we speculate that RVO might be useful as indicator of patients that would still require full-dose DOACs due to an increased arterial thrombotic risk.

There are several limitations to our findings. First and foremost, data are derived from a single center cohort, and although there was very limited loss to follow-up, the outcomes might not be easily generalizable, and the nature of the study does not allow for causal inference. Also, most patients in our cohort used VKAs, while these days DOACs have become the preferred anticoagulant drugs in VTE. Finally, cause of death was unknown in many patients since these details of death records are processed anonymously in the Netherlands. Thus, some episodes of fatal recurrent VTE or arterial events might have been missed. While PTS was only assessed during for a 2-year follow-up, it is known that few cases occur after this time point. 41

A strength of our study is the use of a largely unselected DVT population representing real-life clinical practice, in contrast to other studies that commonly exclude patients with provoked DVT or high recurrence risk. It is particularly within these patient groups that current guidelines mention potential risk differences of clinical relevance. 4 5 6 7 However, this has not yet resulted in differentiation of treatment recommendations, probably due to the lack of evidence to support tailored management in these patient groups. Also, our shared decision-making approach in patients with high D-dimer revealed high refusal rates to resume anticoagulants. This finding emphasizes the strong preference of patients against indefinite anticoagulant therapy and the need for tailored strategies.

In conclusion, our RVO-based management strategy achieved a high proportion of patients that could stop anticoagulation at an overall recurrence rate of 4.4 per 100 patient-years. Since associations of RVO with PTS as well as arterial events were found, it is worth to contemplate RVO-tailored prevention of PTS and intensified cardiovascular risk management in patients with RVO. Moreover, the loss of association of RVO with recurrent VTE in this management study suggests an independent pathogenic relation of RVO with PTS and arterial events. Potential pathogenic mechanisms should be studied to gain insight into modifiable factors that could be used to improve management of DVT patients.

## Notes

**What is known about this topic?**

- RVO has been associated with recurrent VTE and might be used to guide anticoagulant therapy.
- Associations of RVO with other clinical outcomes have also been reported.

**What does this paper add?**

- RVO was not associated with recurrent VTE after extended anticoagulant therapy.
- RVO was associated with post-thrombotic syndrome and arterial events.

## References

[1] Khan F Tritschler T Kahn S R Rodger M A Venous thromboembolism Lancet 2021 398(10294):64 77 33984268 10.1016/S0140-6736(20)32658-1

[2] O'Brien E C Holmes D N Thomas L E Prognostic significance of nuisance bleeding in anticoagulated patients with atrial fibrillation Circulation 2018 138 09 889 897 29678813 10.1161/CIRCULATIONAHA.117.031354

[3] Prins M H Guillemin I Gilet H Scoring and psychometric validation of the Perception of Anticoagulant Treatment Questionnaire (PACT-Q) Health Qual Life Outcomes 2009 7 30 19348685 10.1186/1477-7525-7-30 PMC2686675

[4] Stevens S M Woller S C Baumann Kreuziger L Executive summary: Antithrombotic therapy for VTE disease: Second update of the CHEST guideline and expert panel report Chest 2021 160 06 2247 2259 34352279 10.1016/j.chest.2021.07.056

[5] Esvs Guidelines Committee Kakkos S K Gohel M Baekgaard N Editor's choice - European Society for Vascular Surgery (ESVS) 2021 clinical practice guidelines on the management of venous thrombosis Eur J Vasc Endovasc Surg 2021 61 01 9 82 33334670 10.1016/j.ejvs.2020.09.023

[6] Ortel T L Neumann I Ageno W American Society of Hematology 2020 guidelines for management of venous thromboembolism: treatment of deep vein thrombosis and pulmonary embolism Blood Adv 2020 4 19 4693 4738 33007077 10.1182/bloodadvances.2020001830 PMC7556153

[7] Guideline Committee McCormack T Harrisingh M C Horner D Bewley S Venous thromboembolism in adults: summary of updated NICE guidance on diagnosis, management, and thrombophilia testing BMJ 2020 369 m1565 32430311 10.1136/bmj.m1565

[8] Prins M H Lensing A WA Prandoni P Risk of recurrent venous thromboembolism according to baseline risk factor profiles Blood Adv 2018 2 07 788 796 29632234 10.1182/bloodadvances.2018017160 PMC5894264

[9] RIETE investigators Ageno W Samperiz A Caballero R Duration of anticoagulation after venous thromboembolism in real world clinical practice Thromb Res 2015 135 04 666 672 25708926 10.1016/j.thromres.2015.02.001

[10] Cate V T Lensing A WA Weitz J I Extended anticoagulant therapy in venous thromboembolism: a balanced, fractional factorial, clinical vignette-based study Haematologica 2019 104 10 e474–e477 30846497 10.3324/haematol.2018.209924 PMC6886408

[11] WHITE study group Palareti G Bignamini A Cini M Unprovoked or provoked venous thromboembolism: not the prevalent criterion to decide on anticoagulation extension in clinical practice of various countries-the prospective, international, observational WHITE study Intern Emerg Med 2022 17 01 71 82 34313959 10.1007/s11739-021-02765-1 PMC8313672

[12] Carrier M Rodger M A Wells P S Righini M LE Gal G Residual vein obstruction to predict the risk of recurrent venous thromboembolism in patients with deep vein thrombosis: a systematic review and meta-analysis J Thromb Haemost 2011 9 06 1119 1125 21382171 10.1111/j.1538-7836.2011.04254.x

[13] Tan M Mos I CM Klok F A Huisman M V Residual venous thrombosis as predictive factor for recurrent venous thromboembolism in patients with proximal deep vein thrombosis: a systematic review Br J Haematol 2011 153 02 168 178 21375522 10.1111/j.1365-2141.2011.08578.x

[14] ten Cate-Hoek A J Henke P K Wakefield T W The post thrombotic syndrome: Ignore it and it will come back to bite you Blood Rev 2016 30 02 131 137 26462885 10.1016/j.blre.2015.09.002

[15] Appelen D van Loo E Prins M H Compression therapy for prevention of post-thrombotic syndrome Cochrane Database Syst Rev Wiley 2017 9 09 CD004174 10.1002/14651858.CD004174.pub3 PMC6483721 28950030

[16] Becattini C Vedovati M C Ageno W Dentali F Agnelli G Incidence of arterial cardiovascular events after venous thromboembolism: a systematic review and a meta-analysis J Thromb Haemost 2010 8 05 891 897 20095999 10.1111/j.1538-7836.2010.03777.x

[17] Carrier M Le Gal G Wells P S Fergusson D Ramsay T Rodger M A Systematic review: the Trousseau syndrome revisited: should we screen extensively for cancer in patients with venous thromboembolism? Ann Intern Med 2008 149 05 323 333 18765702 10.7326/0003-4819-149-5-200809020-00007

[18] Dronkers C EA Mol G C Maraziti G Predicting post-thrombotic syndrome with ultrasonographic follow-up after deep vein thrombosis: a systematic review and meta-analysis Thromb Haemost 2018 118 08 1428 1438 29972864 10.1055/s-0038-1666859

[19] Prandoni P Lensing A WA Prins M H The impact of residual thrombosis on the long-term outcome of patients with deep venous thrombosis treated with conventional anticoagulation Semin Thromb Hemost 2015 41 02 133 140 25682083 10.1055/s-0035-1544161

[20] Prandoni P Lensing A WA Prins M H Elastic compression stockings for prevention of the post-thrombotic syndrome in patients with and without residual vein thrombosis and/or popliteal valve reflux[Internet]Haematologica 2022 107 01 303 306 34498448 10.3324/haematol.2021.279680 PMC8719094

[21] Prandoni P Lensing A WA Prins M H Residual venous thrombosis as a predictive factor of recurrent venous thromboembolism Ann Intern Med 2002 137 12 955 960 12484710 10.7326/0003-4819-137-12-200212170-00008

[22] Piovella F Crippa L Barone M Normalization rates of compression ultrasonography in patients with a first episode of deep vein thrombosis of the lower limbs: association with recurrence and new thrombosis Haematologica 2002 87 05 515 522 12010666

[23] Nagler M Ten Cate H Prins M H Ten Cate-Hoek A J Risk factors for recurrence in deep vein thrombosis patients following a tailored anticoagulant treatment incorporating residual vein obstruction Res Pract Thromb Haemost 2018 2 02 299 309 30046732 10.1002/rth2.12079 PMC6055496

[24] Donadini M P Ageno W Antonucci E Prognostic significance of residual venous obstruction in patients with treated unprovoked deep vein thrombosis: a patient-level meta-analysis Thromb Haemost 2014 111 01 172 179 24154729 10.1160/TH13-04-0336

[25] Tan M Bornais C Rodger M Interobserver reliability of compression ultrasound for residual thrombosis after first unprovoked deep vein thrombosis J Thromb Haemost 2012 10 09 1775 1782 22726359 10.1111/j.1538-7836.2012.04827.x

[26] Hassen S Barrellier M T Seinturier C High percentage of non-diagnostic compression ultrasonography results and the diagnosis of ipsilateral recurrent proximal deep vein thrombosis: a rebuttal J Thromb Haemost 2011 9 02 414 418 21070594 10.1111/j.1538-7836.2010.04137.x

[27] Compare 2 Proportions: 2-Sample, 2-Sided Equality [Internet] Statpages.info 2023[cited 2023 Aug 2]. Accessed March 30, 2023 at:https://statpages.info/#Power

[28] DULCIS (D-dimer and ULtrasonography in Combination Italian Study) Investigators Palareti G Cosmi B Legnani C D-dimer to guide the duration of anticoagulation in patients with venous thromboembolism: a management study Blood 2014 124 02 196 203 24879813 10.1182/blood-2014-01-548065

[29] Morgagni Investigators Prandoni P Vedovetto V Ciammaichella M Residual vein thrombosis and serial D-dimer for the long-term management of patients with deep venous thrombosis Thromb Res 2017 154 35 41 28407492 10.1016/j.thromres.2017.04.002

[30] Siragusa S Malato A Anastasio R Residual vein thrombosis to establish duration of anticoagulation after a first episode of deep vein thrombosis: the Duration of Anticoagulation based on Compression UltraSonography (DACUS) study Blood 2008 112 03 511 515 18497320 10.1182/blood-2008-01-131656

[31] AESOPUS Investigators Prandoni P Prins M H Lensing A WA Residual thrombosis on ultrasonography to guide the duration of anticoagulation in patients with deep venous thrombosis: a randomized trial Ann Intern Med 2009 150 09 577 585 19414836 10.7326/0003-4819-150-9-200905050-00003

[32] Meissner M H Zierler B K Bergelin R O Chandler W L Strandness D E Jr Coagulation, fibrinolysis, and recanalization after acute deep venous thrombosis J Vasc Surg 2002 35 02 278 285 11854725 10.1067/mva.2002.121066

[33] Mukhopadhyay S Johnson T A Duru N Fibrinolysis and inflammation in venous thrombus resolution Front Immunol 2019 10 1348 31258531 10.3389/fimmu.2019.01348 PMC6587539

[34] Bouman A C Smits J JM Ten Cate H Ten Cate-Hoek A J Markers of coagulation, fibrinolysis and inflammation in relation to post-thrombotic syndrome J Thromb Haemost 2012 10 08 1532 1538 22642402 10.1111/j.1538-7836.2012.04798.x

[35] Prandoni P Is there a link between venous and arterial thrombosis? A reappraisal Intern Emerg Med 2020 15 01 33 36 31773560 10.1007/s11739-019-02238-6

[36] Robertson L Yeoh S E Ramli A Secondary prevention of recurrent venous thromboembolism after initial oral anticoagulation therapy in patients with unprovoked venous thromboembolism Cochrane Database Syst Rev 2017 12 12 CD011088 29244199 10.1002/14651858.CD011088.pub2 PMC6486093

[37] Noumegni S R Le Mao R de Moreuil C Anticoagulation for VTE: Impact on the risk of major adverse cardiovascular events Chest 2022 162 05 1147 1162 35714709 10.1016/j.chest.2022.05.038

[38] Becattini C Vedovati M C Time to use direct oral anticoagulants to prevent recurrences and major acute cardiovascular events after VTE? Chest 2022 162 05 959 960 36344122 10.1016/j.chest.2022.08.2201

[39] Nicklas J M Gordon A E Henke P K Resolution of deep venous thrombosis: Proposed immune paradigms Int J Mol Sci 2020 21 06 2080 32197363 10.3390/ijms21062080 PMC7139924

[40] Bradbury C Fletcher K Sun Y A randomised controlled trial of extended anticoagulation treatment versus standard treatment for the prevention of recurrent venous thromboembolism (VTE) and post-thrombotic syndrome in patients being treated for a first episode of unprovoked VTE (the ExACT study) Br J Haematol 2020 188 06 962 975 31713863 10.1111/bjh.16275

[41] Schulman S Lindmarker P Holmström M Post-thrombotic syndrome, recurrence, and death 10 years after the first episode of venous thromboembolism treated with warfarin for 6 weeks or 6 months J Thromb Haemost 2006 4 04 734 742 16634738 10.1111/j.1538-7836.2006.01795.x

[42] Bouman A C Atalay S Ten Cate H Ten Wolde M Ten Cate-Hoek A J Biomarkers for post-thrombotic syndrome J Vasc Surg Venous Lymphat Disord 2014 2 01 79 88000 26992975 10.1016/j.jvsv.2013.07.001

[43] Veritas Investigators Prandoni P Ciammaichella M Mumoli N An association between residual vein thrombosis and subclinical atherosclerosis: cross-sectional study Thromb Res 2017 157 16 19 28679112 10.1016/j.thromres.2017.06.036

