# Supplementary material for: Residual Venous Obstruction as an Indicator of Clinical Outcomes following Deep Vein Thrombosis: A Management Study
Source: Thromb Haemost. 2023 Apr 12;123(8):763–72. doi: 10.1055/a-2059-4737 (PMC10365886; doi:10.1055/a-2059-4737)
Supplement: Supplementary file 1 — Supplementary Material [file 10-1055-a-2059-4737-s22080390.pdf]

Supplementary Methods

Study Design

Proximal deep vein thrombosis (DVT) was defined as thrombosis in the popliteal, femoral, and/or common femoral vein. Patient characteristics were defined in a previous publication.[1] While there were no exclusion criteria, patients with DVT complicated by pulmonary embolism, patients with cancer, or those who followed further treatment at other institutions usually did not end up in the clinical care pathway (CCP). Iliofemoral DVT was defined as thrombosis present in the common femoral vein at diagnosis by ultrasound examination. Venous insufficiency was defined according to the CEAP classification (Clinical Etiological Anatomical Pathophysiological) as a score C2 or higher.[2] Definitions of transient provoking factors were in agreement with those proposed by the International Society of Thrombosis and Haemostasis (ISTH).[3] Factors had to be present within 2 months before DVT diagnosis.

Management Strategy

All patients received at least 6 months of elastic compressive therapy. Antiplatelet drugs were discontinued upon DVT diagnosis and restarted at cessation of anticoagulant therapy.

D-Dimer Measurement

Venous blood samples were collected in commercially available tubes and centrifuged according to recent guidelines. D-dimer levels were determined in a certified laboratory at Maastricht University Medical Center (MUMC) using established methods as previously described.[1] Levels were measured in citrated plasma using the Vidas assay (bioMérieux Clinical Diagnostics, Marcy-l'Etoile, France) until May 2008 and the Innovance assay (Siemens Healthcare, Marburg, Germany) from June 2008 onwards. The laboratory cutoff value of 500 ng/mL was used to define high D-dimer.

Clinical Outcomes

The Villalta score was assessed at each visit during the CCP. Post-thrombotic syndrome was defined according to the original definition by Villalta.[4] Types or arterial events were classified in accordance with standardized definitions.[5] Cancer was diagnosed with appropriate diagnostic methods in response to clinical suspicion following routine clinical examination or through national screening programs. Basal or squamous cell skin cancer were not included since their registration tends to be less complete and less accurate. Patients with cancer at baseline were considered not at risk for cancer during follow-up. Major bleeding events while on anticoagulant therapy as defined by ISTH were recorded at each visit during the CCP and through hospital records afterwards.[6]

Statistical Analysis

Patient characteristics were included in the multivariable model of a certain clinical outcome if they were present in more than 75% of bootstrapped models produced by backward stepwise logistic regression. This was performed using R version 3.5.3.[7]

References

1 Nagler M, Ten Cate H, Prins MH, Ten Cate-Hoek AJ. Risk factors for recurrence in deep vein thrombosis patients following a tailored anticoagulant treatment incorporating residual vein obstruction. Res Pract Thromb Haemost 2018;2(02):299–309

2 Eklöf B, Rutherford RB, Bergan JJ, et al;American Venous Forum International Ad Hoc Committee for Revision of the CEAP Classification. Revision of the CEAP classification for chronic venous disorders: consensus statement. J Vasc Surg 2004;40(06):1248–1252

3 Kearon C, Ageno W, Cannegieter SC, Cosmi B, Geersing GJ, Kyrle PASubcommittees on Control of Anticoagulation, and Predictive and Diagnostic Variables in Thrombotic Disease. Categorization of patients as having provoked or unprovoked venous thromboembolism: guidance from the SSC of ISTH. J Thromb Haemost 2016; 14(07):1480–1483

4 Villalta S, Bagatella P, Piccioli A, Lensing A, Prins M, Prandoni P. Assessment of validity and reproducibility of a clinical scale for the post-thrombotic syndrome. J Vasc Surg Venous Lymphat Disord 2014;2(01):8–14

5 Hicks KA, Mahaffey KW, Mehran R, et al;Standardized Data Collection for Cardiovascular Trials Initiative (SCTI) 2017 cardiovascular and stroke endpoint definitions for clinical trials. Circulation 2018;137(09):961–972

6 Schulman S, Kearon CSubcommittee on Control of Anticoagulation of the Scientific and Standardization Committee of the International Society on Thrombosis and Haemostasis. Definition of major bleeding in clinical investigations of antihemostatic medicinal products in non-surgical patients. J Thromb Haemost 2005;3(04):692–694

6 R Core Team. R: A language and environment for statistical computing. R Foundation for Statistical Computing, Vienna, Austria. 2014 at: http://www.R-project.org/

 © 2023. The Author(s).
